#  Kangfuxin alleviates ulcerative colitis in rats by inhibiting NF-κB p65 activation and regulating T lymphocyte subsets

**DOI:** 10.22038/IJBMS.2023.68771.14990

**Published:** 2023

**Authors:** Miao He, Wan-xin Yu, Yongmei Shen, Jing-na Zhang, Lian-li Ni, Yue Li, Heng Liu, Yu Zhao, Hai-rong Zhao, Cheng-gui Zhang

**Affiliations:** 1 Yunnan Provincial Key Laboratory of Entomological Biopharmaceutical R&D, College of Pharmacy, Dali University, Dali, China; 2 Yunnan Provincial 2011 Collaborative Innovation Center for Entomoceutics, Dali, Yunnan, China; 3 School of Pharmacy, Shanghai Jiao Tong University, Shanghai, China; 4 Good Doctor Pharmaceutical Group, Chengduo, Sichuang, China; 5 Cancer and Anticancer Drug Research Center, School of Pharmaceutical Sciences, Wenzhou Medical University, Wenzhou, Zhejiang, China; # These authors contributed eqully to this work

**Keywords:** Inflammation mediators, Inflammatory bowel disease, Kangfuxin, T lymphocyte subsets, Ulcerative colitis

## Abstract

**Objective(s):**

Ulcerative colitis (UC) remains an enduring, idiopathic inflammatory bowel disease marked by persistent mucosal inflammation initiating from the rectum and extending in a proximal direction. An ethanol extract of *Periplaneta americana *L., namely Kangfuxin (KFX), has a significant historical presence in Traditional Chinese Medicine and has been broadly utilized in clinical practice for the treatment of injury. Here, we aimed to determine the effect of KFX on 2,4,6-trinitro’benzene sulfonic acid (TNBS)-induced UC in Sprague-Dawley rats.

**Materials and Methods:**

We established the UC model by TNBS/ethanol method. Then, the rats were subject to KFX (50, 100, 200 mg/kg/day) for 2 weeks by intragastric gavage. The body weight, disease activity index (DAI), colonic mucosal injury index (CMDI), and histopathological score were evaluated. The colonic tissue interleukin (IL)-1β, IL-6, tumor necrosis factor-α (TNF-α), IL-10, transforming growth factor-1 (TGF-β1), and epidermal growth factor (EGF) were determined by Elisa. To study T-lymphocyte subsets, flow cytometry was performed. In addition, the expression level of NF-κB p65 was evaluated by immunohistochemistry and western blot analysis.

**Results:**

Compared with the TNBS-triggered colitis rats, the treatment of rats with KFX significantly increased the body weight, and decreased DAI, CMDI, and histopathological score. Also, KFX elicited a reduction in the secretion of colonic pro-inflammatory cytokines, namely IL-1β, IL-6, and TNF-α, concomitant with up-regulation of IL-10, TGF-β1, and EGF levels. Upon KFX treatment, the CD3+CD4+/CD3+CD8+ ratio in the spleen decreased, while the CD3+CD8+ subset and the CD3+CD4+CD25+/CD3+CD4+ ratio demonstrated an increase. In addition, the expression of NF-κB p65 in the colon was decreased.

**Conclusion:**

KFX effectively suppresses TNBS-induced colitis by inhibiting the activation of NF-κB p65 and regulating the ratio of CD4+/CD8+.

## Introduction

Ulcerative colitis (UC), majorly affecting the colon and rectum, is an inflammatory bowel disease (IBD) linked to the immune system (1, 2). The etiology of UC is multifaceted, encompassing an array of intricate factors such as aberrant cytokine secretion, dysregulated immune responses, altered barrier function, and modified intestinal microbiota (3, 4). The immune response and inflammatory pathogenesis of UC demonstrate that tissue injury results from complex and dynamic interactions between cells and cytokines. Diverse cell types, such as antigen-presenting cells (dendritic cells and macrophages), T helper cells, regulatory T cells, and natural killer T cells, significantly contribute to UC’s pathology by regulating inflammation. Particularly, the pathogenesis of UC has been associated with a lack or dysfunction of CD3^+^CD4^+^CD25^+ ^T cells (regulatory T cell, Treg) (5, 6).

The predominant proportion of mature T cells express either CD4 or CD8αβ glycoprotein, which allows for their categorization into 2 subsets, namely CD4+ and CD8+(7, 8*)*, and they have been both identified in the peripheral blood and intestinal mucosa of adult or pediatric patients with IBD during inflammatory episodes (9-11). Usually, CD4^+^ T cells generate pro-inflammatory cytokines in immune responses to mediate inflammation. The activation of CD4^+^ T cells is controlled by CD8^+^ T cells which also regulate mucosal immunity (12). Meanwhile, the disordered immune function activates the NF-κB signaling, which further enhances the release of pro-inflammatory mediators (13). Though anti-inflammatory agents such as glucocorticoids, salicylates, and corticosteroids show therapeutic effects, there is no certain cure for UC yet.

Kangfuxin (KFX), an extract obtained from *Periplaneta americana *L*.* (Blattidae), has received approval from the China Food and Drug Administration (CFDA) (Z51021834). The primary constituents of KFX comprise nucleotides, amino acids, and oligopeptides. KFX has been reported to exhibit a multitude of therapeutic attributes, such as tissue regeneration and wound healing (14), with a notable focus on gastric and duodenal ulcers. Moreover, KFX is an effective agent against diabetic foot ulcers and reflux esophagitis induced by lysolecithin and hydrochloric acid (15). Recently, clinical investigations have demonstrated the therapeutic potential of KFX in the management of gastrointestinal ulcers *(*16-18*)* and UC (19*)*. However, the exact mechanism of KFX in 2,4,6-trinitrobenzene sulfonic acid (TNBS)-induced UC is unclear. Therefore, this study was designed to assess the therapeutic efficacy of KFX in TNBS-induced UC in rats. 

## Materials and Methods

***Preparation and properties of KFX***

KFX was prepared as described in patent CN100525781. To elaborate, 200 g dry powder of *P. americana* L. was first subjected to two rounds of 75% ethanol extraction (1.2 kg and 0.8 kg, respectively) at 70 °C for 8 hr. Subsequently, the resulting filtrates are mixed and concentrated to attain a density of 1.13-1.16 g/ml through rotary evaporation at 70 °C. The residue was added to 3 times (v/w) 70% ethanol for repeated extraction at 70 °C for 6 hr. Next, the filtrates were mixed and condensed by a rotary evaporator at 70 °C until attaining a relative density of 1.09–1.16 g/ml. Post evaporation, the extract was added to water and stirred at 70 ℃ for 1 hr and then allowed to stand for 12 hr. The oil was removed, and the solution was collected, filtered, and condensed to a density of 1.20 g/ml via rotary evaporation at 70 °C. The sample was concentrated to 1.16–1.22 g/ml by rotary evaporation at 70 °C. Lastly, 50 ml glycerin was added to each 1000 ml medicine solution, stirred evenly, and filtered. Then, the filtrate was boiled for 30 min, packed separately, and sterilized at 125 ℃, 0.1 Mpa, for another 30 min. Upon cooling to room temperature, the final KFX preparation was obtained.

The properties of KFX were analyzed according to the method described in the invention patent CN106370739A. HPLC was conducted utilizing an Agilent 1260 instrument (Agilent, USA). The separation of KFX constituents was achieved by employing an Agilent Reversed-phase C18 alkyl silica gel column (Agilent, USA), integrated with a preconnected in-line column filter, and maintained at a temperature of 35 °C. The amino acid contents of KFX were determined by Nihhydrin colorimetry assay using L-alanine as a standard. 

***Animals***

All animal procedures and handling were approved by the Institutional Animal Care and Use Committee (IACUC) of Dali University, China (Animal Ethics No. DLU2017-0117). The rats were housed in a specific-pathogen free (SPF) facility under controlled conditions of temperature (18–22 °C), humidity (48–52%), and a 12-hr light/dark cycle with *ad libitum* access to food and water. Adult Sprague-Dawley rats of both genders weighing between 180–220 g were procured from Hunan Silaike Jingda Experimental Animal Co, Ltd. (License: SCXK (Xiang) 2016-0002), and the experimental protocol was approved under permit certificate SYXK (Dian)-2018-0002. The animals were maintained in well-ventilated plastic cages with woodchip bedding.

***Induction of colitis and drug treatment***

Colitis was triggered by TNBS (SLBP0889V, Sigma, USA) as described previously *(*20*)*. Briefly, rats were anesthetized and placed in a prostrate position. Polypropylene tubes with a diameter of approximately 1.5–2 mm were positioned in an inverted suspension state and carefully introduced into the colonic region of rats via the anus, with a depth of insertion ranging from 7–9 cm. The injection of TNBS (25 mg/kg in 30% ethanol) was rapidly administered while the air was simultaneously injected to ensure complete delivery of the solution to the intestinal lumen via the polypropylene tube. To maintain the position of the solution, the anus was firmly pinched and kept upside-down for a duration of 1 min. The occurrence of hematochezia or positive occult blood in rats indicated the successful establishment of the UC model. Similarly, for the normal control group, rats received normal saline instead of TNBS.

To establish the UC model in rats, drug treatments and assessments were performed as per the method depicted in Figure 2A. The rats were graded for the degree of inflammation after 7 days of TNBS-induced UC as reflected by disease activity index (DAI) *(*21*)* (Table 1). Following ulcer severity assessment, the rats were then randomly allocated into six distinct groups, comprising normal control group, model group: TNBS, positive group: TNBS+ Salazosulfapyridine (SASP) (a sulfonamides antimicrobial drug, 300 mg/kg, i.g.), and TNBS+KFX (50, 100, 200 mg/kg, *i.g.*) groups (KFX, Z51021834, Good Doctor Pharmaceutical Group, Sichuan, China). Rats in the normal control group were treated similarly without TNBS. After UC was established, rats were treated with drugs (SASP or KFX, respectively) as indicated from the 8^th^ day until day 21. The DAI, body weight, stool consistency, and the presence of occult blood were measured on days 7, 10, 14, and 21 after UC. 

At 21 days, rats were sacrificed to dissect the liver, spleen, lung, kidney, colon, and thymus. These were weighed for calculating the respective organ index. The colon was flushed with cold normal saline and then cut longitudinally for the morphological studies. 

***Morphological evaluation***

Rats were euthanized, and their colons were promptly harvested to evaluate colonic length and weight, colon mucosal damage index (CMDI), and histopathological score. The CMDI was calculated as follows (22, 23*)*:

a score of 0 indicates no damage; a score of 1 reflects mucosal congestion, edema, erosion, or ulceration; a score of 2 relates to mucosal congestion, edema, mucous membrane roughness, mild erosion, or intestinal adhesion; a score of 3 signifies mucosal congestion, edema, moderate erosion, and ulcer formation, with an ulcer diameter of less than 1 cm; and a score of 4 characterizes mucosal congestion, edema, moderate erosion, and ulcer formation, with an ulcer diameter greater than 1 cm.

Colon tissue samples were subjected to histological studies. Briefly, collected colon tissue samples were fixed with 4% paraformaldehyde (24) for 12–24 hr, dehydrated, and embedded in paraffin. Subsequently, the colon coronal sections (5 µm) were cut using a rotatory microtome (Leica RM2245, Germany) and stained with hematoxylin and eosin (H&E). Colon damage was assessed as described previously (Table 2) (25, 26*)*.

***Enzyme-linked immunosorbent assay (ELISA)***

A 10% colonic homogenate was prepared in normal saline and centrifuged to obtain the supernatant, which was then stored at -80 °C. ELISA was performed on the 10% colonic homogenate for TNF-α, IL-1β, IL-6, IL-10, EGF, and TGF-β1 following the manufacturer’s instructions. Briefly, the standards and samples were added to the wells, and biotin-conjugated antibodies were introduced, followed by washing. Avidin-conjugated HRP was added and substrate solution was introduced, resulting in color development. The absorbance of the color was quantified.

***Detection of the subtypes of T lymphocytes in the spleen***

Splenic parts were minced and then rubbed over a 70-μm gauze. Single-cell suspensions were collected by centrifugation. The supernatant was removed and erythrocytes in the spleen were lysed with 1.0 ml Gey’s solution. Then, the cells were washed twice with 3% fetal calf serum-phosphate buffer (FBS-PBS). Then, the cells were re-suspended in 3% FBS-PBS, and the cell density was adjusted to 1×10^7^/ml. The cells were subjected to triple washing using buffer solution (PBS supplemented with 0.5% bovine serum albumin and 0.02% sodium azide), followed by staining with fluorochrome-conjugated monoclonal antibodies: CD3+-FIFC (Cat#553062, BD Bioscience), CD4+-APC (Cat# 553062, BD Bioscience), CD8+-PE (Cat#553062, BD Bioscience), CD25+-PE (Cat#553062, BD Bioscience). Samples were analyzed using Flow Cytometry (CytoFLEX S). Subsequent data analysis was carried out with FlowJo software (Tree Star Inc, USA). 

***Immunohistochemistry analysis***

For immunohistochemistry, the colon sections were sequentially incubated with primary antibody against NF-κB p65 (1:200) and HRP-conjugated secondary antibody. Positive staining was visualized using chromogen diaminobenzidine (DAB, lot, No. 1709305920, Fuzhou Maixin Biotechnology Development Co. LTD, China). Finally, hematoxylin was used for counterstaining for 3 min. NF-κB p65 proteins turned positive for brown-yellow staining in the cell membrane and cytoplasm. Three different horizons of each slice were randomly selected and photographed under a microscope. After selecting the region of interest, the NF-κB p65 protein levels were measured by Image Pro® Plus 60 software. After calculating the IOD Sum and the Area Sum, the average optical density value MOD (MOD = IOD Sum/Area Sum) was determined to estimate the expression of positive cells.

***Immunoblotting***

Briefly, RIPA buffer containing protease and phosphatase inhibitors was used to effectively lyse the colon tissue samples and extract total protein. Aliquots were then electrophoretically separated by SDS-PAGE and transferred onto PVDF membranes, which were probed with specific antibodies, including β-actin, NF-κB p65 (8242S, CST, USA), p-NF-κB p65 (Ser536) (76778, CST, USA), and p65 (ab16502, Abcam, UK) antibodies, to detect the target proteins. Following washing, the signals were detected using an enhanced chemiluminescence detection system (Thermo, USA) and acquired by the Imaging system (Azure Biosystems C280, USA). Quantification of western blotting data was executed via the ImageJ software. For western blotting, three random samples were selected from each group.

***Statistical analysis***

Statistical analyses were conducted using SPSS 21.0 or GraphPad Prism 8. The normality of the data was assessed using the Kolmogorov-Smirnov test. One-way ANOVA followed by Dunnett’s test or Student’s t-test was used for multiple group comparisons with normally distributed data. The Kruskal-Wallis test was used for non-normally distributed data. Statistical significance was defined as *P*<0.05. Data are presented as mean±SEM or SD.

## Results

***Determination of major chemical components in KFX***

**Figure 1:**
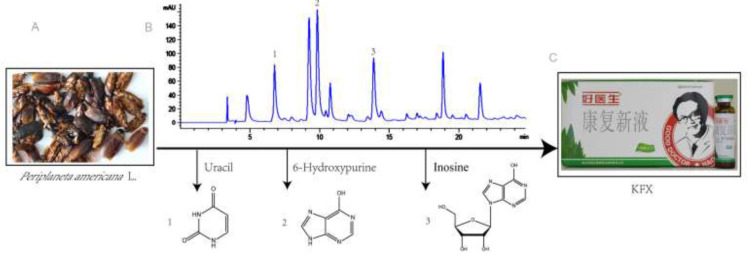
Content determination of the main chemical compounds in KFX

KFX is a brown solution. It smells aromatic, slightly fishy, and tastes bitter salty. As shown in Figure 1, HPLC analysis revealed that the three major components in KFX extract are uracil (9.25 g/ml), hypoxanthine (21.97 g/ml), and inosine (25 g/ml). In addition, using Nihhydrin colorimetry, we also found that the total amino acid content in KFX was 0.748 mg/ml. This was in accordance with the revised quality standard of KFX issued by the State Drug Administration (WS3-B-3674-2000 (Z) which states that the total amino acid content in KFX should be > 0.72 mg/ml. 

***KFX reduces the disease activity index and alters the immune organ index***

**Figure 2:**
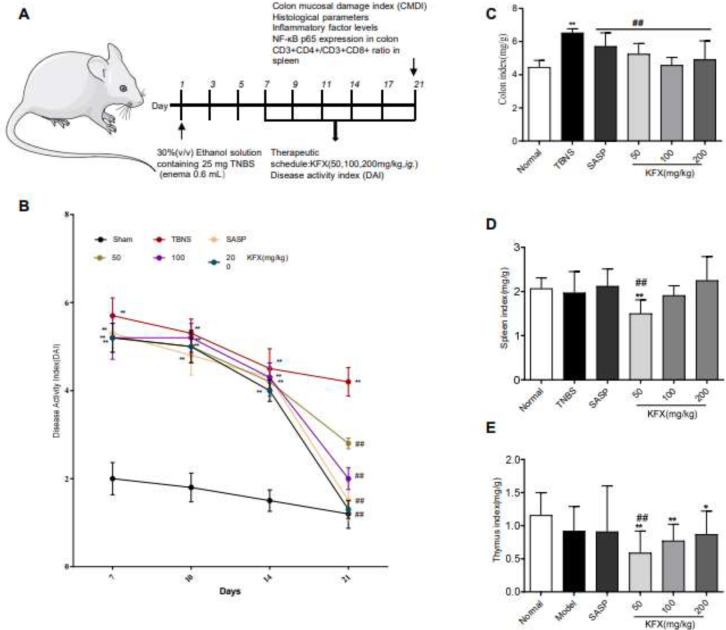
Effect of KFX on disease activity index (DAI) and immune organ index in rats with TNBS-induced colitis

DAI score represents a practical and efficient method for assessing the degree of inflammation and severity of colitis. *(*27, 28*)*. After UC, all model rats exhibited anorexia, poor spirit, weight loss, and loose/bloody stools. On the 7^th^ day, in the model rats, the DAI score was significantly increased by the presence of TNBS but was dramatically decreased by KFX treatment (Figure 2B). Notably, at 21 days, compared with the vehicle group, the DAI score in SASP and KFX groups decreased markedly (*P*<0.01) (Figure 2B). Interestingly, compared with the sham group, DAI in the SASP or KFX (200 mg/kg) groups shows no significant difference (Figure 2B).

Regarding the organ index, in comparison with the sham group, in the TNBS group colon index significantly surged (*P*<0.01) (Figure 2C), while the spleen and thymus indexes decreased (Figure 2D-E). Notably, compared with the TNBS group, the colon index showed a marked decrease in KFX (50,100,200 mg/kg)-treated rats (*P*<0.01) (Figure 2C). Also, in the KFX group, the dose-dependent increase of the spleen and thymus indexes was observed.

***KFX improves intestinal mucosa injury in UC rats***

**Figure 3:**
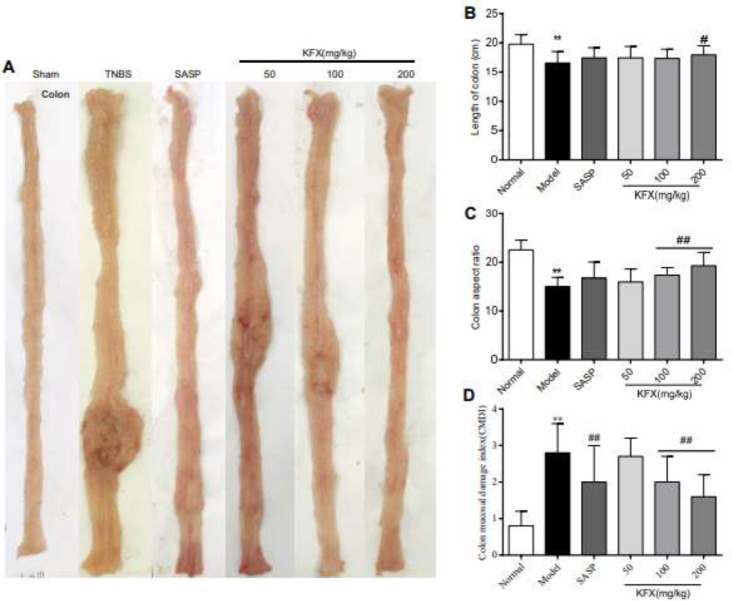
Effect of KFX on colon length, length-width ratio of the colon, and colon mucosal damage index (CMDI) in rat with TNBS-induced colitis

As shown in Figure 3A, the colon in the sham group displayed no edema, ulcer, or erosion, whereas, the colon in the TNBS group showed evident congestion, edema, multiple ulcers, and erosion. These changes were noticed mostly in the distal part of the colon and got further severe towards the proximal anal end. However, as shown in Figure 3B-D, TNBS treatment shortened the colon length, decreased the length-width ratio, and increased CMDI (*P*<0.01). Additionally, mucosal injury showed clear signs of improvements in the KFX (100, 200 mg/kg) group with improved colon length, length-width ratio, and CMDI (*P*<0.01).

***KFX modulates the cytokine profile in the colon tissue of rats with TNBS-induced colitis***

**Figure 4.**
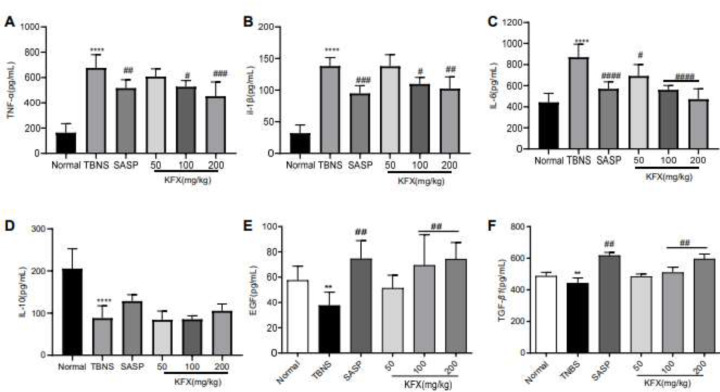
Effects of KFX on colonic IL1-β, IL-6, TNF-α, IL-10, EGF, and TGF-β1 in rats with TNBS-induced colitis

Cytokines play pivotal roles in the pathogenesis of UC *(*4*)*. Since we found that KFX improved intestinal mucosa inflammation in UC rats, the levels of associated cytokines in the colon were measured. The pro-inflammatory cytokines IL-6, IL-1β, and TNF-α, which are primarily secreted by macrophages, play a crucial role in the pathogenesis of colonic mucosal inflammation and the consequent disruption of the mucosal barrier. (29*)*. In comparison with the sham group, the TNBS group showed elevated levels of IL-6, IL-1β, and TNF-α (Figure 4A-C), but IL-10, EGF, and TGF-β1 levels were reduced (*P*<0.01) (Figure 4D-F). In contrast, these were reversed upon treatment with KFX (100 or 200 mg/kg) (Figure 4A-C).

***KFX improves inflammatory cell infiltration and epithelial cell damage***

**Figure 5:**
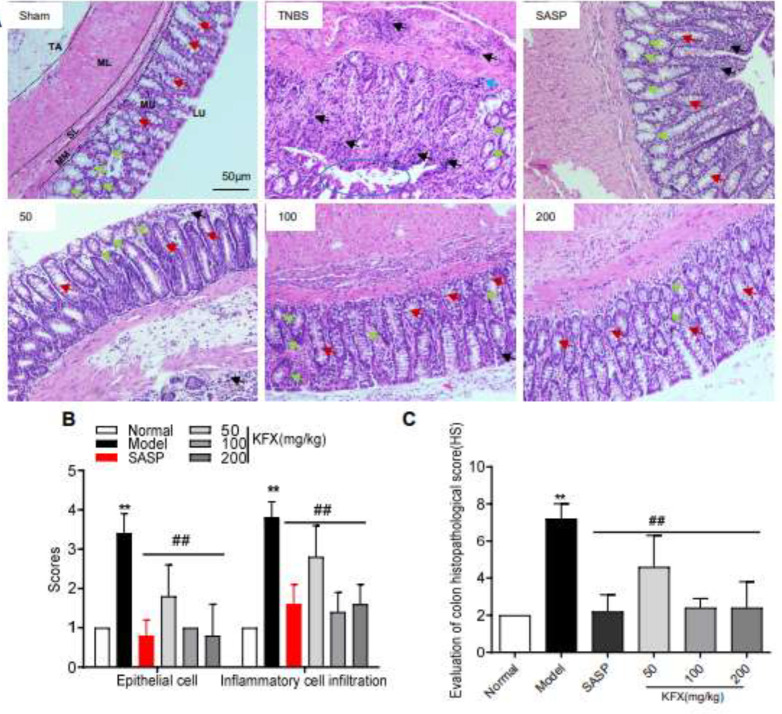
Effects of KFX on colon histology in rats with TNBS-induced colitis

Microscopic observations revealed that in the control group, the submucosal, muscularis, and adventitial layers of the colonic mucosa exhibited an exquisite structural organization, the mucosal epithelium appeared intact and the goblet cells were clearly discernible. Notably, no evidence of inflammatory cell infiltration was observed. Histological examination of the sham group demonstrated absence of inflammatory cell infiltration, whereas the colon tissues of TNBS-induced rats exhibited notable crypt destruction, mucosal ulceration, and a substantial influx of inflammatory cells. However, the administration of KFX resulted in reduced histopathological abnormalities, as evidenced by decreased colon tissue damage observed in HE staining (Figure 5A). KFX significantly reduced histological score, which was determined by evaluating the combined severity of inflammatory cell infiltration and mucosal damage in TNBS-induced bowel disease (Figure 5B-C). These findings suggest that KFX may have a therapeutic potential in ameliorating TNBS-induced bowel disease in rats.

***KFX mitigates the expression of NF-κB p65 in the colon***

**Figure 6:**
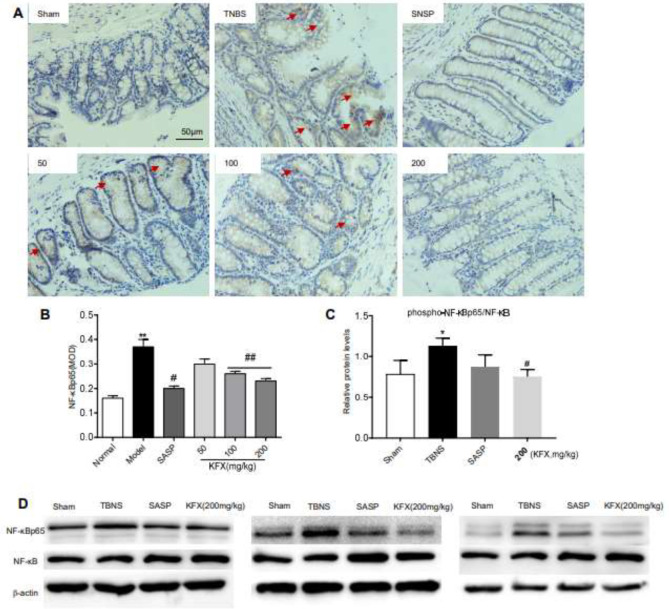
KFX inhibits NF-κB p65 protein expression in the colon

NF-κB signaling has been implicated in the pathogenesis and progression of colitis. Compared with the sham group, immunohistochemical analysis revealed a significant increase in the level of NF-κB p65 in the colonic tissues of the TNBS-induced rats. (Figure 6A). KFX treatment (100, 200 mg/kg) significantly decreased NF-κB p65 level in rats (Figure 6A-B), especially in the cell nucleus and cytoplasm of colonic mucosa. Importantly, similar results were also obtained using immunoblotting. Comparably, KFX (200 mg/kg) reduced the level of NF-κB p65 in the colon (Figure 6C-D). These findings suggest a plausible association between the regenerative effects of KFX in UC and the suppression of NF-κB p65 activation.

***KFX modulates*** ***the CD4***^+^***/CD8***^+^*** ratio in UC ***

**Figure 7:**
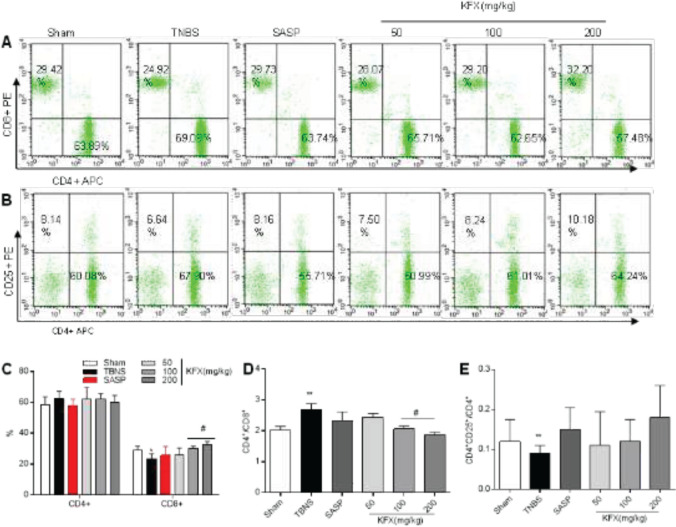
Effects of KFX on T cell subtypes in the spleen in rats with TNBS-induced colitis

CD3^+^CD8^+^ T lymphocytes decreased in the model group compared with the sham group. However, the CD3^+^CD4^+^ T lymphocytes/CD3^+^CD8^+^ T lymphocytes ratio was increased. Moreover, the CD3^+^CD4^+^CD25^+^/CD3^+^CD4^+^ ratio was decreased (*P*<0.01) (Figure 7A-E). In comparison with the TNBS group, in the KFX groups (100 or 200 mg/kg), elevation of CD3^+^CD8^+^ was observed (*P*<0.05) (Figure 7C). Also, CD3^+^CD4^+^/CD3^+^CD8^+ ^T ratio decreased (Figure 7D). Likewise, CD3^+^CD4^+^CD25^+^/CD3^+^CD4^+^ ratio and CD3^+^CD4^+^CD25^+^/CD3^+^CD4^+^ T lymphocytes was decreased (*P*<0.01 or *P*<0.05) too (Figure 7A-E). 

## Discussion

UC and IBD represent clinicopathological features of colonic mucosa inflammation. It is known that an imbalanced mucosal immune system triggers the release of inflammatory mediators, causing chronic inflammation, ulceration, edema, and lesions in the colonic mucosa (30-32). Long-term UC leads to electrolyte imbalance, tachycardia, and marasmus resulting in poor quality of life. In this study, firstly, we determined the major chemical contents of KFX extract, which was also validated for quality against the revised quality standard issued by the State Drug Administration (WS3-B-3674-2000 (Z)). Then, we observed that KFX treatment significantly ameliorated the symptoms of TNBS-induced colitis. Based on these findings, it is plausible to suggest that KFX may hold potential as a therapeutic intervention for the treatment of UC.

*P. americana *(33), commonly known as the American cockroach, has a substantial record of clinical utilization. Some also reported that *P. americana* could modulate the ratio of T and B lymphocytes (34). Previously, we showed that the ethanol extract of *P. Americana* (KFX liquid) produced therapeutic effects against oxazolone (OXZ)-induced acute UC in rats *(*35*)*, and the ethanol extract of *P. americana* L. alleviates UC triggered by a combination of chronic stress and TNBS in rats (36). Our findings also supported the dramatic efficacy of KFX on the symptoms of UC rats.

KFX down-regulates the levels of inflammatory cytokines, such as IL-1β, IL-6, and TNF-α levels induced by TNBS. Additionally, the levels of TGF-β1, IL10, or EGF were increased by KFX treatment. UC is characterized by an inflammatory response that leads to up-regulation in the production of pro-inflammatory cytokines, including TNF-α, IL-6, and IL-1β, ultimately causing tissue damage and disease progression (37). TNF-α, in particular, plays a crucial role in the pathogenesis of IBD. TNF-α-targeting agents are commonly employed in the treatment of patients with active UC. Macrophages produce TNF-α in large quantities in response to pro-inflammatory processes, and cytokine performs various functions in the development of colitis. Notably, TGF-β1, a potent anti-inflammatory cytokine suppresses inflammatory responses, promotes cellular proliferation, modulates cell growth and differentiation, and enhances immunity (38). Here, we observed that compared with the TNBS group, a significant decrease in IL-1β, IL-6, and TNF-α level was observed once UC rats were treated with different doses of KFX or SASP, while TGF-β1 and IL-10 level was increased. These findings suggest that KFX is capable of modulating the immunological equilibrium between pro-inflammatory and anti-inflammatory cytokines. Also, EGF is an inducer of cellular growth, migration, tissue proliferation, and repair of great importance in preserving the structural and functional integrity of intestinal mucosa (39). Interestingly, the reduced level of EGF in the TNBS group also increased upon treatment with KFX, suggesting another protective mechanism of KFX in promoting the repair of the injured colon mucosa.

The present study also revealed that KFX effectively mitigated rat colitis by repressing overexpressed NF-kB. NF-κB is a multifaceted modulator of numerous genes that participate in inflammatory responses*(*40). Therefore, inhibiting NF-κB activation and the release of cytokines have become a major focus in UC research for the treatment of intestinal inflammation (41). The nuclear translocation and activation of NF-κB represent crucial stages in the release of inflammatory cytokines, thereby instigating the inflammatory response. (42). Likewise, our findings demonstrated that KFX ameliorated UC by modulating this pathway, thereby corroborating the significance of NF-kB in the advancement and pathophysiology of inflammatory disorders, including UC. TNBS colitis is primarily a model for Crohn’s disease (CD)(43-45). In TNBS-induced colitis, a genetic association between NOD2 and inflammation has been demonstrated, which is absent in UC. Given these findings, the expression of autophagy-associated genes implicated in the etiology of CD should have been investigated (46). Nevertheless, the exact impacts of KFX on UC or CD, as well as the plausible mechanisms, necessitate further investigation.

CD3 serves as a marker for mature T lymphocytes, with CD3^+^ T lymphocytes being classified into CD4^+^ helper T lymphocytes and CD8^+^ suppressor/cytotoxic T lymphocytes. The CD3^+^CD4^+^ helper T lymphocytes participate in phagocytosis-mediated anti-infective defense mechanisms and B cell-mediated humoral immune responses. Moreover, CD3^+^CD8^+^ T cells exhibit target-specific direct cytotoxicity against cells (47). Additionally, Treg T cells, a subtype of CD4+ T cells, are responsible for maintaining immune homeostasis, reducing inflammation, and inducing immune tolerance. (48). Furthermore, compared with the sham group, the UC model group showed a lower percentage of lymphocytes and CD4^+^CD25^+^ regulatory T cells while the ratio of CD3^+^CD4^+^ T lymphocytes and CD3^+^CD8^+^ T lymphocytes increased. This feature of UC is also in accordance with the findings of Postovalova’s group (49). In contrast, in the spleen of UC rats, different doses of KFX and SASP increased the proportion of T lymphocytes and CD4^+^CD25^+^ regulatory T cells and reduced CD3^+^CD4^+^/ CD3^+^CD8^+ ^T cell ratio. This indicated the modulatory effects of KFX in balancing the T lymphocyte subtypes and the Th/Ts ratio to alleviate immune responses and inflammatory injuries.

**Table 1:** The scoring criteria of disease activity index for ulcerative colitis

Score	Weight loss (%)	Stool consistency	Fecal occult blood
0	normal or weight increase	normal	negative
1	≥ 0 % and ≤ 5 %		
2	> 5 % and ≤ 10 %	loose stools	positive
3	> 10 % and ≤ 15 %		
4	> 15 %	Watery diarrhea	gross bleeding

Note: DAI Score = Weight loss + Stool consistency + Fecal occult blood

**Table 2:** The criteria of histologic score for ulcerative colitis

Score	Epithelium	Infiltration
0	normal morphology	no infiltrate
1	loss of goblet cells	infiltrate around crypt basis
2	loss of goblet cells in large areas	infiltrate reaching to Lamina muscularis mucosae
3	loss of crypts	Extensive infiltration reaching the Lamina muscularis mucosae and thickening of the mucosa with abundant edema
4	loss of crypts in large areas	infiltration of the Lamina submucosa

(A) The dried insect body of *Periplaneta americana* L. (B) HPLC chromatogram is shown. Each numbered peak represents a specific chemical compound. The main compounds were measured as follows: 1) uracil 9.25 g/ml; 2) hypoxanthine 21.97 g/ml; 3) inosine 25 g/ml. (C) KFX liquid

KFX: Kangfuxin

(A) Induction of colitis, KFX-treated and assessment timeline are illustrated schematically. DAI scores were evaluated and recorded at 7, 10, 14, and 21 days after treatment with TNBS. (B) Rats were treated with drugs (normal saline, SASP, or KFX) for 14 days. (C) Colon index. (D) Spleen index. (E) Thymus index. Data were presented as mean±SD (n =6-8 per group). *, ** represent statistical significance between normal control versus other groups at *P*<0.05, *P*<0.01 levels; #, ##represent statistical significance between model control versus other groups at *P*<0.05, *P*<0.01 levels (full text is consistent)

KFX: Kangfuxin; TNBS: 2,4,6-trinitrobenzene sulfonic acid; SASP: Salazosulfapyridine

(A) Morphology of colon in rats. (B) The length of the colon. (C)Length-width ratio of the colon. (D) CMDI scores of each group were determined. Data were presented as mean±SD (n = 6–8per group)

KFX: Kangfuxin

Release of IL1-β, IL-6, TNF-α, IL-10, EGF, and TGF-β1 in the colon was determined by ELISA. The expression level of TNF-α (A), IL1-β (B), IL-6 (C), IL-10 (D), TGF-β1 (E), and EGF (F). Data were presented as mean±SD (n = 6–8 per group)

KFX: Kangfuxin; TNBS: 2,4,6-trinitrobenzene sulfonic acid

(A) Microscopic observation of H&E staining. TNBS-induced rats presented marked crypt destruction, mucosal ulceration, and significant inflammatory cell infiltration. (B) Epithelial cell scores and inflammatory cell infiltration were determined. (C) Histopathological scores (HS) of each group were determined. LU=Lumen, MU=Mucosa layer, MM=Muscularis mucosa, SL=Submucosa layer, ML=Muscle layer, TA=Tunica adventitia. Red arrow represents the goblet cell. Green arrow represents the crypt. Black arrow represents inflammatory infiltration. Blue arrow represents granulation tissue. Blue circle represents necrotic tissue shedding. Scale bars:50 µm. Data were presented as mean±SD (n = 6–8 per group)

KFX: Kangfuxin; TNBS: 2,4,6-trinitrobenzene sulfonic acid

(A) Representative immunohistochemistry (IHC) for NF-κB p65 in UC rats. (B) Quantification of NF-κB p65. (C-D) Phosphorylated levels of NF-κB p65 in the colon of UC mice. Data are presented as mean±SD (n = 6 per group). Data were presented as mean±SD (n = 3–6 per group). Scale bars: 50 µm

KFX: Kangfuxin; TNBS: 2,4,6-trinitrobenzene sulfonic acid; SASP: Salazosulfapyridine

(A) CD3+CD4+ T lymphocytes and CD3+CD8+ T lymphocytes. (B) CD3+CD4+CD25+ T lymphocytes. (C) CD3+CD4+ T lymphocytes and CD3+CD8+ T lymphocytes were quantified. (D) CD3+CD4+/CD3+CD8+ ratio. (E) CD3+CD4+CD25+/ CD3+CD4+ ratio. Data were presented as mean±SD (n = 4 per group)

KFX: Kangfuxin; TNBS: 2,4,6-trinitrobenzene sulfonic acid; SASP: Salazosulfapyridine

## Conclusion

Collectively, we established a colitis model induced by TNBS and elucidated the impact of KFX on the progression of UC. Our results substantiated that KFX ameliorated intestinal tissue damage in the rat model and suppressed the inflammatory response in the intestinal tissues of rats. Additionally, we observed that KFX modulated the activation of TNBS-induced NF-κB p65 and T lymphocyte subsets, thereby impeding the progression of UC. Consequently, KFX exhibits potential as a viable therapeutic agent for the management of UC.

## Authors’ Contributions

Z CG and Z HR conceived the study; Z JN, H M, and Y WX performed data curation; H M performed formal analysis; Z HR and Z CG helped procure funding; L H and Z Y carried out investigation; H Y, Z JN, L Y, and N LL provided methodology; Z CG was responsible for project administration; L H provided resources; Z HR provided validation; H M helped with visualization; Z HR and H M helped write the original draft; Z CG was responsible for writing the review and editing.

## Data Availability

The data that was utilized to corroborate the outcomes of this research have been incorporated within the manuscript and can be freely accessed.

## Conflicts of Interest

None.
